# Patient-centered endpoints in trials of ICU sedation

**DOI:** 10.1186/s13054-014-0536-7

**Published:** 2014-09-29

**Authors:** Jorge IF Salluh, Robert D Stevens

**Affiliations:** D’Or Institute for Research and Education, Rua Diniz Cordeiro, 30 Botafogo, Rio de Janeiro, 22281-100 RJ Brazil; Praça Cruz Vermelha, 23, Centro 20230-130, Rio de Janeiro, RJ Brazil; Division of Neurosciences Critical Care, The Johns Hopkins University School of Medicine, 733 North Broadway, Baltimore, MD 21205 USA

We read with interest the study by Zhou and colleagues because it represents an important effort towards advancing the knowledge in current sedation strategies [1]. However, one crucial issue in sedation trials is to choose clinically relevant and patient-centered endpoints.

Recent randomized trials demonstrate that sedation strategies with similar short-term mortality rates and ICU length of stay may still be associated with different short-term and long-term cognitive outcomes [2,3]. Limiting outcome evaluation to hospital mortality or length of stay may fail to capture important treatment effects. With converging evidence that survival of critically ill patients is rising, mortality is becoming an increasingly insensitive way to measure the efficacy of an intervention.

Multiple studies demonstrate that ICU delirium is a prevalent syndrome that can be detected with simple validated diagnostic tools [4,5]. ICU-acquired weakness is identified in nearly one-half of patients with sepsis, multiorgan failure, or protracted mechanical ventilation [6]. ICU-based trials need to focus on survivors and on the evaluation of other outcome domains such as delirium, long-term cognitive function, psychological status, muscle strength, functional status, and quality of life [3]. These endpoints are particularly relevant in designing trials of sedation strategies.
